# The Interaction between Oligodendrocytes and Aβ in Alzheimer's Disease

**DOI:** 10.2174/0115672050401966250625171338

**Published:** 2025-07-07

**Authors:** Wenjing Wang, Xueyan Huang, Zucai Xu, Changyin Yu

**Affiliations:** 1 Department of Neurology, Affiliated Hospital of Zunyi Medical University, Zunyi, 563000, China;; 2 Guizhou Provincial Key Laboratory of Brain Function and Prevention and Treatment of Brain Disease, Zunyi, 563000, China

**Keywords:** Alzheimer's disease, oligodendrocytes, myelin, Aβ, neurodegenerative disorder, central nervous system (CNS)

## Abstract

Oligodendrocytes (OLs) are the primary myelinating cells in the central nervous system (CNS), responsible for maintaining the rapid conduction of nerve signals and ensuring neuronal stability through metabolic and nutritional support. Recent studies have reported that OLs are also involved in the development and progression of Alzheimer's disease (AD), particularly in the production and clearance of amyloid-beta (Aβ), exhibiting complex and critical regulatory functions. While traditional research has predominantly focused on the roles of neurons and microglia in Aβ metabolism, recent evidence indicates that OLs engage in a complex bidirectional interaction with Aβ in AD. On the one hand, OLs can produce Aβ, frequently generating aggregated and highly toxic Aβ_42_, which contributes to plaque expansion and disease progression. On the other hand, neuron-derived Aβ exerts a concentration-dependent dual effect on OLs. At high concentrations, it induces oxidative stress and cell apoptosis, while at low concentrations, it promotes their differentiation and myelin repair functions. Therefore, OLs serve as both a “source” and a “target” of Aβ production and response, making them a key factor in AD pathogenesis. This review discusses the interaction between OLs and Aβ in AD, aiming to provide new perspectives on targeting OLs for AD therapy. Given the dual role of OLs in Aβ metabolism, targeting OLs dysfunction and the regulatory mechanisms underlying Aβ production and clearance could provide novel therapeutic strategies for AD. Future research should investigate the roles of specific OL populations (including oligodendrocyte precursor cells (OPCs), pre-myelinating OLs, and mature OLs) in Aβ generation and metabolism, focusing on the signaling pathways involved. Additionally, the molecular mechanisms by which OLs regulate other glial cells, such as astrocytes and microglia, through intercellular signaling to facilitate Aβ clearance and maintain neuroglial homeostasis warrant further exploration.

## 1 INTRODUCTION

Alzheimer's disease (AD) is a progressive neurodegenerative disorder characterized by cognitive loss, memory impairment, language difficulties, and behavioral changes. The pathological process of AD is characterized by neuronal damage and loss, which are closely linked to amyloid-beta (Aβ) deposition and abnormal tau protein phosphorylation [1]. For a long time, studies on AD have focused on neuronal injury and its associated pathological changes [2-4], but in recent years, the importance of glial cells, especially oligodendrocytes (OLs), has been gradually revealed [5-7]. As myelin-forming cells in the central nervous system (CNS), OLs not only play a crucial role in the formation and maintenance of myelin sheaths, ensuring the rapid transmission of neural signals [8], but also provide essential metabolic support and nutrient supply to neurons [9-11].

Studies have reported that OLs are deeply implicated in the pathological progression of AD and are closely involved in the production, accumulation, and clearance of Aβ [12]. Aβ accumulation is considered to be one of the core pathological features of AD, and OLs may play a key role in the generation, transport and clearance of Aβ [12]. In addition, OLs may become dysfunctional in the early stages of AD, even before classical pathological changes, such as amyloid plaques and neurofibrillary tangles (NFTs)[13-14]. Therefore, a comprehensive understanding of the interaction between OLs and Aβ will contribute to elucidating the early pathological mechanisms of AD and provide promising new directions for therapeutic development.

## 2 BIBLIOGRAPHIC APPROACH

A comprehensive literature search was conducted in the Web of Science (WOS) database using keywords pertaining to “oligodendrocytes” and ” amyloid-beta “, with no restriction on the publication year, resulting in a total of 487 relevant records. The search was limited to English language publications, and document types were restricted to articles and reviews. Proceedings papers, early access, meeting abstracts, and book chapters were excluded. Additional exclusion criteria were applied to eliminate studies that were not relevant to the research topic. Based on the inclusion criteria, a total of 460 eligible articles were ultimately included as the study sample. The detailed search strategy and screening process are illustrated in Fig. (**1**). The limited number of articles indicates that the association between OLs and Aβ remains insufficiently explored and warrants further investigation.

## 3 FUNCTIONS OF OLs AND THEIR ALTERATIONS IN AD

### 3.1 Structure and Function of OLs

**Fig. (2):**
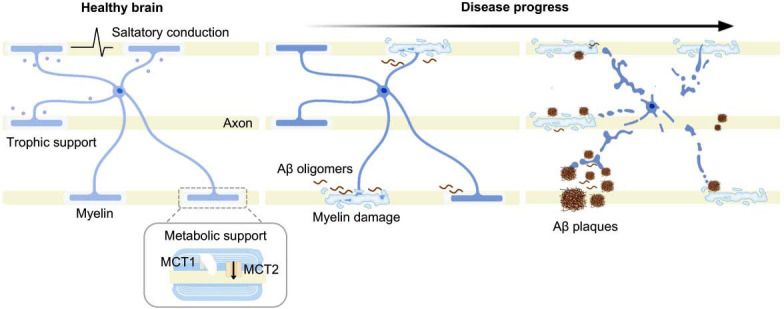
Dynamic changes in OLs during the progression of AD. In healthy conditions, OLs facilitate saltatory conduction of neural electrical signals through myelin formation and provide metabolic and nutritional support to neurons. During the progression of AD, OLs are vulnerable to pathological factors and exhibit distinct cellular phenotypes in response to the continuously evolving microenvironment. In the early stages, mild exposure to Aβ induces significant alterations in OLs gene expression, accompanied by initial impairment of myelin function. As the disease advances and Aβ burden increases, OLs dysfunction and myelin loss become more pronounced, alongside the emergence of specific OLs subpopulations expressing C4b and Serpina3n.

OLs are one of the most abundant and vital glial cell types in the CNS, accounting for about 75% of the total glial population [15]. They have compact cell bodies and extensively branched processes, which allow them to myelinate multiple axons simultaneously by forming multilayered, concentric myelin sheaths [16]. This distinctive structure enables OLs to efficiently produce myelin, facilitating rapid and precise transmission of neural signals [16] (Fig. **2**). The morphology of OLs varies significantly depending on brain region, axon diameter, and their specific functional requirements [17]. For example, Type I OLs, found in both gray and white matter, possess numerous processes suited for myelinating many small-diameter axons. Type II OLs, limited to white matter, feature polygonal cell bodies and fewer processes. Types III and IV, mainly in the spinal cord, have larger cell bodies and longer processes, specializing in myelinating large-diameter or long-range axons [18, 19]. This diversity reflects OLs’ adaptability and their key role in regulating myelination, which is vital for CNS structure and function [16]. OLs derive from oligodendrocyte precursor cells (OPCs), the most proliferative cells in the adult CNS, which retain lifelong potential to proliferate, differentiate, and mature into myelinating OLs [20, 21]. New OLs continue to integrate into mature neural circuits throughout adulthood, supporting myelin formation and repair, and thereby maintaining myelin plasticity [22, 23]. In addition to myelination, OLs play a vital role in supporting neuronal metabolism, particularly in maintaining axonal energy homeostasis [24]. They produce lactate, which is transported to axons *via* monocarboxylate transporters (MCT1 and MCT2) highly expressed in myelin, where it is converted into ATP to meet axonal energy demands [25, 26] (Fig. **2**). Notably, Munyeshyaka *et al.* demonstrated that OLs and their precursors contribute critically to memory processes, such as consolidation and associative recall [27]. In summary, through their specialized structure, regenerative capacity, and metabolic support, OLs play critical roles in myelination, axonal maintenance, neural circuit stability, and cognitive function.

### 3.2 Functional Alterations of OLs in AD

Single-cell transcriptomics analysis showed that OLs exhibit pathologic responsiveness in addition to neurons and microglia, revealing their important role in AD [28-31]. It was found that in postmortem brain samples from AD patients with TREM2 mutations, differentiation genes of OLs were down-regulated, while genes related to lipid metabolism and oxidative stress were elevated, suggesting that OLs undergo metabolic adaptation reflecting compensatory responses to impaired myelin formation and neuronal degeneration [32]. Although most AD animal model studies have focused on neuronal and protein lesions, some have also revealed functional defects in OLs, suggesting that OLs may play a crucial role in the pathogenesis of AD [33-36]. Single-cell transcriptomic analysis of the 5×FAD mouse model revealed a novel population of OLs responsive to Aβ accumulation, exhibiting specific gene expression features, such as the complement component C4b and the serine protease inhibitor Serpina3n, indicating altered OLs responsiveness in AD [32] (Fig. **2**). A population called “disease-associated OLs” has been detected in different models of neurodegenerative pathologies [6, 37, 38]. However, the activation pattern of OLs in AD differs significantly from that in other neurodegenerative diseases, suggesting that OLs are more influenced by Aβ in AD, highlighting the distinctiveness of AD pathology [38]. Recent spatial transcriptomics studies have confirmed that the expression of myelin-rich and OL-related genes in the brains of mice with AD-associated mutations is altered early during Aβ exposure, with significant downregulation in areas of intensive Aβ accumulation [28]. Thus, OLs are essential in the pathogenesis of AD and may also play key roles in its early pathological changes, providing a potential basis for future early diagnosis and therapeutic targets.

## 4 HETEROGENEITY OF OL-DERIVED AND NEURON-DERIVED Aβ IN AD

Traditionally, Aβ was considered to be primarily produced by neurons [39]. However, with the increasing research on OLs, it has become progressively clearer that OLs also contribute to the production and accumulation of Aβ [12, 40]. The unique Aβ-generating capacity of OLs in glial cells provides a new perspective on the pathogenesis of AD. Both RNA sequencing [41] and proteomics data [42] have shown that all key genes required for Aβ generation are expressed in OLs, including amyloid precursor protein (APP), beta secretase (BACE1), and components of γ-secretase. Further analysis using RNAscope *in situ* hybridization revealed that approximately 80% of OLs in layers V and VI of the cortex in patients with sporadic Alzheimer's disease (sAD) co-express both APP and BACE1 [43]. In addition, contrary to the traditional belief that the loss of OLs is associated with myelin degeneration [35], the number of OLs in layers V and VI of the prefrontal cortex of the brain in patients with sAD was instead increased, suggesting that the role of OLs in AD may go beyond the mere function of myelin formation or repair, thus indicating a close link especially in the production and accumulation of Aβ [43]. Further studies have demonstrated that the expression levels of APP and BACE1 in OLs are significantly elevated and more stable compared to those in neurons [43]. More importantly, through comparative analysis, it was found that human induced pluripotent stem cell (iPSC)-derived OLs produced more Aβ than neurons in the same cell line, indicating that OLs may be more efficient in Aβ production, and this efficiency was verified in cell lines with different genotypes [43-45]. Therefore, the source of Aβ is not limited to neurons; OLs are also capable of producing Aβ, and the Aβ production from OLs is more efficient and stable compared to neurons.

Aβ variants from different sources exhibit significant differences in structure and aggregation properties, with Aβ produced by OLs and neurons characterized by distinct mechanisms of generation and deposition patterns. It has been shown that the thickness of the cell membrane and the type of organization affect the function of γ-secretase, which determines the type and proportion of Aβ [46]. γ-Secretase is a multi-subunit transmembrane enzyme complex essential for the processing of APP, particularly in the generation of Aβ peptides. The key components of this enzyme complex include Presenilin-1 (PSEN1), Presenilin-2 (PSEN2), Nicastrin (NCSTN), APH1, and PSENEN. These subunits work together in concert to cleave APP and produce Aβ peptides [47]. In OLs, the specificity of the lipid composition plays an important role in the production of Aβ. The cell membranes of OLs are enriched with cholesterol, glycolipids, and phospholipids of long-chain and saturated fatty acids, which results in a denser and thicker membrane structure [48]. This membrane density may provide more substrate for γ-secretase, which leads to an increase in the Aβ_42/40_ ratio, and this elevated ratio correlates with an increased aggregation capacity of Aβ, which, in turn, promotes the formation of Aβ oligomers and protofibrils [12]. OLs are more inclined to generate a higher proportion of Aβ_42_ due to their unique lipid environment, and these Aβ_42_ aggregates are more likely to form oligomers and protofibrils with toxicity. As a result, Aβ generated by OLs exhibits a stronger profile in terms of aggregation and toxicity.

Interestingly, studies have reported that as key components of the γ-secretase complex, the expression profiles of PSEN1 and PSEN2 differ significantly between OLs and neurons. PSEN1 is expressed at a much higher level in OLs compared to neurons, while PSEN2 is expressed at relatively lower levels in OLs [43]. Although PSEN1 and PSEN2 have some functional overlap within the γ-secretase complex, their distinct roles in Aβ generation and ratio regulation may account for the differences in Aβ characteristics between OLs and neurons [49]. PSEN1 plays a dominant role in the regulation of Aβ production, and its mutations typically lead to a significant increase in Aβ_42_ generation. For example, in the PSEN1^-/-^ mouse model, APP cleavage is notably inhibited, although it is not completely abolished, indicating the critical role of PSEN1 in APP processing [50]. Although PSEN2 can partially compensate for the function of PSEN1 in certain contexts, such as during embryonic development and in specific neuronal types [51], PSEN1 remains the primary mediator of Aβ production [52]. In the presence of PSEN1, PSEN2 is not required for γ-secretase function, further reinforcing the dominant role of PSEN1 in Aβ generation [52]. In contrast, in neurons from PSEN2 knockout mice, Aβ production was found to be similar to that of normal neurons, indicating that PSEN2 has a minor impact on APP processing. However, functional mutations in PSEN2 typically enhance Aβ_42_ production through a gain-of-function mechanism [52]. Therefore, it can be hypothesized that the elevated expression of PSEN1 in OLs is a key factor underlying its dominant role in the generation of Aβ_42_. Given the relatively low expression of PSEN2 in OLs, this may render OLs more dependent on PSEN1, thereby contributing to an enhanced production of Aβ_42_ during amyloidogenesis.

Rajani and colleagues further demonstrated the toxic heterogeneity of OL-derived Aβ in AD by differentiating OLs from iPSCs of familial AD patients and healthy controls [43]. Although OLs generate large amounts of Aβ_42_, this does not imply that amyloid plaques will be predominantly deposited in the white matter. In fact, the number of amyloid plaques in white matter is significantly less than in gray matter [53, 54]. This phenomenon may be attributed to the lower synaptic density in white matter, which limits the local accumulation of Aβ and makes it difficult to reach the threshold for plaque formation [55]. At the same time, microglia and astrocytes in white matter may effectively inhibit abnormal Aβ aggregation through efficient clearance mechanisms and homeostatic regulatory functions [56-58]. However, the interplay between Aβ derived from OLs in white matter and the clearance mechanisms mediated by other glial cells remains unclear. Therefore, a comprehensive investigation of the interactions between OLs and other glial cells in AD, along with their regulatory roles in Aβ production and clearance, is of great significance.

## 5 DUAL EFFECTS OF NEURON-DERIVED Aβ ON OLs: INJURY AND PROTECTION

### 5.1 Aβ Toxicity Damages OLs

**Fig. (3):**
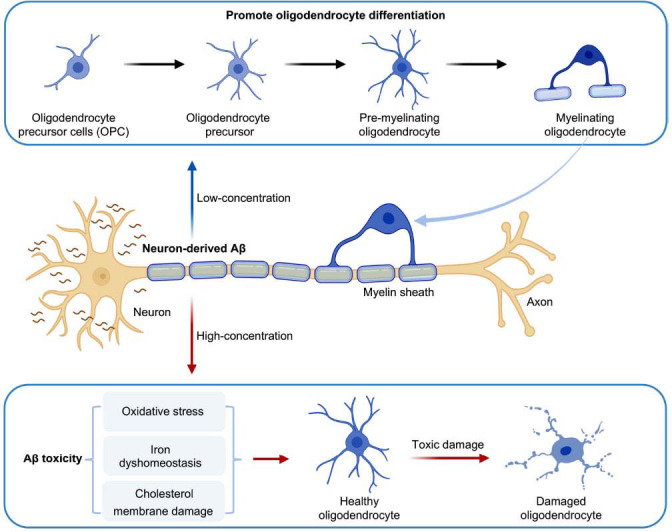
Neuron-derived Aβ exhibits a complex and dual regulatory effect on OLs. High levels of Aβ accumulation compromise OLs' function and viability *via* multiple pathological pathways, including oxidative stress, iron dyshomeostasis, and disruption of cholesterol-rich membrane domains, ultimately resulting in demyelination and impaired neural conduction. In contrast, low concentrations of Aβ oligomers demonstrate potential protective and supportive effects by promoting OLs' survival, enhancing the differentiation and maturation of OPCs, and facilitating the formation and repair of new myelin.

In the white matter of AD patients, the accumulation of high levels of Aβ not only damages neurons but may also lead to the death of OLs [59]. Aβ poses a serious threat to the survival of OLs through multiple mechanisms, including oxidative stress, increased iron loading, and cholesterol membrane damage [60-62] (Fig. **3**). OLs are particularly vulnerable to Aβ-induced oxidative stress due to their low reduced glutathione levels and high iron content, which weakens their antioxidant defense and increases their susceptibility to oxidative damage [63, 64]. Oxidative stress promotes cell death by damaging intracellular lipids, proteins, and DNA, which, in turn, promotes cell death [65]. In addition, Aβ disrupts cholesterol-rich membranes, including those of OLs and their myelin sheaths, impairing membrane structure and function, ultimately leading to cell death [66]. Although Aβ plaques are relatively rare in AD white matter, the level of soluble Aβ oligomers in white matter is significantly elevated, implying that OLs in white matter may be directly exposed to high concentrations of Aβ [67]. Aβ is indeed toxic to OLs, but clinical treatments aimed at removing Aβ plaques have not been effective in stopping neurodegeneration and cognitive decline in AD patients [68, 69]. This suggests that the toxic effect of Aβ may not only exert its influence through plaque accumulation, but it may also affect white matter through other mechanisms. In addition, Aβ activates neutral sphingomyelinase (nSMase) by inducing oxidative stress, which, in turn, promotes ceramide production as a key mechanism for its induction of OLs' death [70]. As a key second messenger in oxidative stress-induced apoptosis, ceramide plays a central role in Aβ-induced cell death. Aβ-induced oxidative stress damages mitochondrial DNA and activates transcription factors like NF-κB and AP-1, which enhance nSMase activity and further promote ceramide production and cell death [70]. Notably, the cytotoxic effects of Aβ_1-40_ and Aβ_25-35_ on OLs have been confirmed by several studies [71, 72]. In particular, the cytotoxic effects of Aβ_1-40_ and Aβ_25-35_ were particularly evident in OLs expressing intermediate differentiation markers [73]. In addition, the toxicity of Aβ_25-35_ on OLs has also been demonstrated [70]. Studies have shown that the cytotoxic effect of Aβ peptide is most pronounced when its concentration exceeds 20 μM, which may be closely related to the cellular stress response triggered by the high concentration of Aβ peptide and its interference with key intracellular molecules and signaling pathways [74]. However, the toxic effects of different Aβ fragments on OLs vary, with Aβ_1-42_ exhibiting stronger cytotoxicity in mature OLs. This may be attributed to the heightened sensitivity of mature OLs to Aβ, along with the increased amyloid deposition and fibrillization characteristics of Aβ_1-42_. Upon exposure to Aβ_1-42_, OLs exhibited significant morphological changes, such as protrusion disruption and cell body contraction, which are usually hallmarks of apoptosis, suggesting that Aβ_1-42_ may activate apoptotic signaling pathways [74]. In addition, Aβ_1-42_ triggered the release of lactate dehydrogenase by disrupting the cell membrane, further exacerbating the cell death process [73].

### 5.2 Aβ Promotes OLs Differentiation and Myelin Repair

The conventional view is that Aβ has a negative effect on OLs [75, 76]; however, recent studies have found that different molecular forms of Aβ, as well as the concentration and duration of exposure, may lead to completely different cellular responses [77]. These differences may arise from the distinct mechanisms of neurotoxicity mediated by Aβ monomers and oligomers, especially in the complex pathologic context of AD, where multiple forms of Aβ may affect the function of OLs in different ways [78]. A study using a 3×Tg-AD mouse model noted that although Aβ accumulation was associated with dysfunction in OLs, it did not directly lead to the death of OLs. Instead, an increase in the myelin marker myelin basic protein (MBP) as well as an increase in the number of CC1^+^ OLs in hippocampal tissues of 6- to 18-month-old mice indicated that OLs may undergo a reparative response under the influence of Aβ oligomers [72]. This suggests that Aβ oligomers may regulate myelin synthesis by influencing the repair mechanism of OLs rather than directly inducing cell death. Future research should explore the mechanisms by which different forms of Aβ affect OLs, with a particular focus on the impact of low Aβ concentrations. Additionally, studies should investigate the repair mechanisms of OLs, examining how changes in local Aβ concentration influence OL function and whether Aβ regulates myelin synthesis by altering OL differentiation or repair processes. In addition, Aβ oligomers in OLs regulate the survival, differentiation and myelin synthesis of OLs by interacting with the adhesion protein integrin β1 (Itgb1), which activates Fyn kinase and CaMKII [79, 80]. Aβ oligomers, through the activation of Fyn kinase, promote the local translation of the 18.5 kDa isoform of MBP [81], which is expressed predominantly in OLs and is essential in the process of myelin formation [79]. More importantly, Aβ oligomers also enhanced myelin regeneration and OLs lineage restoration in an injury model, suggesting that Aβ may contribute to the pathological environment of AD by promoting the repair function of OLs, rather than simply inducing cell death [65, 77] (Fig. **3**). Furthermore, inhibiting Fyn kinase was able to significantly block the Aβ-induced increase in OLs' survival and differentiation process, indicating a central role for Fyn in the functional regulation of OLs [79]. By exploring the functional link between Aβ oligomers and Fyn kinase-mediated differentiation, maturation, and survival of OLs, we revealed the complex role of Aβ oligomers in OLs, particularly their dual effect on myelin sheath formation, which can both promote repair and cause damage at high concentrations. This also highlights Fyn kinase as a potential therapeutic target [82, 83].

## 6 ROLE OF OL-DERIVED Aβ IN AD

**Fig. (4):**
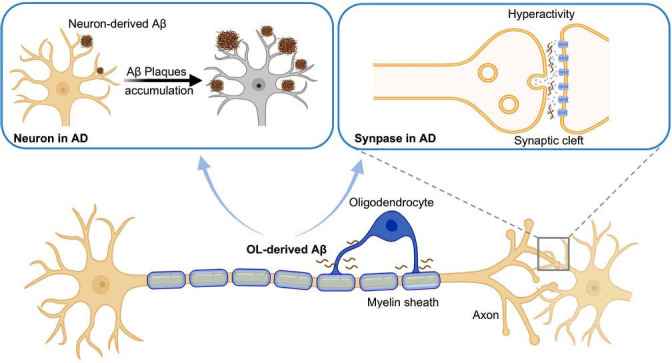
OL-derived Aβ exerts a dual influence on amyloid pathology and neuronal function. It facilitates the propagation and enlargement of amyloid plaques initially seeded by neuron-derived Aβ, thereby accelerating pathological Aβ accumulation. Simultaneously, OL-derived Aβ increases neuronal excitability, driving aberrant hyperactivity that contributes to early neural network dysfunction in AD.

By constructing an AD mouse model with OLs deficient in BACE1, it was found that BACE1-deficient mice had significantly fewer Aβ plaques in the visual cortex, post-splenial cortex, and motor cortex compared to controls, particularly in cortical layers V and VI, a region highly susceptible to early plaque deposition and cognitive decline in AD [84]. It was further found that despite the presence of BACE1 in OLs, BACE1 deficiency in excitatory neurons almost completely eliminated amyloid plaques (95-98% reduction), a result that contrasts with the effects produced by OL-derived Aβ [85]. It is reasonable to speculate that neuron-derived Aβ may trigger the early accumulation of plaques at higher levels, whereas OL-derived Aβ is more likely to promote plaque growth and expansion. This finding highlights the unique role of OLs in Aβ generation, particularly in the early stages of Aβ plaque formation. While neuron-produced Aβ initiates the initial accumulation of plaques at higher levels, OL-derived Aβ is more likely to promote plaque growth and expansion, further exacerbating the pathology of AD [86] (Fig. **4**). Due to their specific role and location in the nervous system, OLs may act as a bridge in the early stages of AD by modulating Aβ production, accumulation, and clearance. This process not only facilitates plaque formation but also contributes to the progression of subsequent pathology.

Furthermore, BACE1 knockdown in OLs effectively eliminated early neuronal abnormal activity in App^NL-G-F^ mice [87], suggesting that Aβ production by OLs is a potential cause of early neuronal dysfunction in AD [43]. In particular, despite the suppression of Aβ generation in OLs, neuronal firing patterns and temporal structure were restored to levels similar to those of wild-type control mice [43]. This implies that Aβ generated in OLs impacts neuronal activity and contributes directly to the early onset of neurological dysfunction in AD [88]. It was also demonstrated that local field potential and neuronal responses to CA1 sharp ripple events were not significantly affected in BACE1 knockout mice during electrophysiological activities in the cortico-hippocampal network, which further supports the role of OLs-generated Aβ in maintaining the electrophysiological integrity of the cortico-hippocampal network [43, 89, 90]. Despite the suppression of Aβ accumulation in neurons, OL-derived Aβ appears to contribute to the phenomenon of neuronal hyperactivity in early AD by influencing neuronal firing activity (Fig. **4**). This reveals that the generation of Aβ in OLs may promote the phenomenon of neuronal hyperactivity in early AD by influencing neuronal firing activity [43]. In addition, it was further demonstrated that even without Aβ produced by other cells, OL-derived Aβ itself was sufficient to trigger neuronal hyperactivity, further validating the close association between OL-derived Aβ and abnormal neuronal firing [85]. Thus, Aβ generated by OLs is not only a promoter of plaque formation, but may also contribute to AD-related early neurological dysfunction by affecting neuronal activity.

## 7 POTENTIAL THERAPEUTIC STRATEGIES FOR AD TARGETING OLs

As research on AD progresses, an increasing number of drugs and treatments have been proposed, aiming to repair OLs' function or promote myelin regeneration, slowing the progression of AD and improving cognitive deficits [91-93]. For example, donepezil improves cognitive function [94] and promotes remyelination by stimulating the differentiation of neural stem cells into OLs [95]; solanezumab can also promote the differentiation and myelination of OLs [96], and their combination significantly enhances the cognitive benefits of donepezil [97]. Moreover, it has been found that fluoxetine can delay the aging of the OLs spectrum and promote their maturation, thus improving the function of OLs in the hippocampus [98]. In addition to classical pharmacotherapy, a series of novel therapeutic strategies have emerged in recent years. For example, allopregnanolone has been shown to promote the differentiation of OLs and exhibit significant potential in AD treatment [99]. In addition, 27-hydroxycholesterol, a small molecule, has shown positive effects on OLs maturation and myelin formation. This small molecule is expected to restore damaged neural networks by regulating the developmental process of OLs, thereby enhancing cognitive function [100]. As for plant extracts, *Gardenia jasminoides J. Ellis* extract promotes the differentiation of OPCs by inhibiting neuroinflammation, enhances the maturation of OLs, and ameliorates myelin sheath damage, highlighting the potential of phytotherapy in AD [101]. Recent studies have found that exercise therapy helps to restore the differentiation and maturation of OLs, thereby ameliorating myelin damage [102]. In particular, physical therapy targeting OLs has also shown great potential in AD treatment. For example, low-intensity pulsed ultrasound therapy has been shown to be effective in repairing damaged OLs in AD [103].

The therapeutic strategy of targeting OL-derived Aβ based on the interaction between OLs and Aβ provides a new research perspective for AD [12]. It was found that by knocking down BACE1 in OLs, the early neuronal hyperactivity phenotype triggered by soluble Aβ in App^NL-G-F^ mice could be effectively eliminated, and the neuronal firing level and temporal structure could be restored [43]. The firing rate of neurons was also significantly increased by injecting Aβ aggregates containing OLs' conditioned medium [43]. In addition, Aβ deposition can be significantly reduced by targeting knockdown of BACE1 in OLs, and the effects of this approach are comparable to the efficacy of FDA-approved Aβ antibodies [104, 105]. In particular, selective targeting of BACE1 in OLs avoids the side effects associated with widespread inhibition of BACE1, especially the adverse effects seen in the use of clinical BACE1 inhibitors [106-108]. In addition, single-cell RNA sequencing studies revealed that OLs expressing several key molecules, including ADAM10, Ano4, IL-33, ApoE, and Sort1, were significantly upregulated in a mouse model targeting OLs deficient in BACE1 [84]. ADAM10 inhibited Aβ production through cleavage of APP, whereas Ano4 was able to regulate the activity of ADAM10 [109], suggesting that BACE1-deficient OLs may promote non-amyloid pathways and reduce Aβ production by upregulating ADAM10 and Ano4. Meanwhile, higher expression of ApoE, an Aβ scavenging factor, contributes to the reduction of Aβ accumulation [110, 111]. BACE1 deletion may indirectly affect Aβ production and clearance by regulating molecules, such as ApoE.

Studies have demonstrated that selective deletion of BACE1 in OLs significantly reduces Aβ production and alleviates amyloid plaque deposition, but may also indirectly modulate Aβ clearance and metabolism by altering interglial signaling dynamics [84]. Astrocytes actively participate in the clearance and degradation of Aβ and exhibit a certain degree of resistance to Aβ toxicity through their metabolic activities, such as glycolysis [112, 113]. When Aβ secretion by OLs decreases, astrocytes may detect alterations in Aβ levels or associated metabolic signals and subsequently activate feedback mechanisms to preserve local homeostasis [84]. Moreover, microglia exhibit high sensitivity to fluctuations in Aβ concentrations and the surrounding metabolic milieu. Under conditions of decreased Aβ, they migrate toward lesion sites and increase their phagocytic activity, thereby facilitating enhanced Aβ clearance [114, 115]. Notably, inflammatory mediators secreted by activated microglia can reciprocally modulate OLs' function, establishing a bidirectional regulatory mechanism [116, 117]. Developing intervention strategies that coordinate OLs and their interactions with microglia and astrocytes holds great potential to suppress Aβ production and enhance its clearance, thereby improving cerebral Aβ homeostasis. Compared to approaches targeting a single cell type, this multicellular strategy may provide greater efficacy and specificity in slowing AD progression.

## CONCLUSION

This review highlights the role of OLs in AD, emphasizing their contribution to Aβ production and regulation of its metabolism. Future studies should focus on the specific cell types responsible for Aβ production in the spectrum of OLs (OPCs, pre-myelinating oligodendrocytes, myelinating oligodendrocytes) and explore how OLs regulate Aβ metabolism through specific pathways in pathological states. Moreover, it is crucial to elucidate the molecular mechanisms by which oligodendrocytes participate in Aβ metabolism, particularly whether they indirectly regulate the functions of other glial cells, such as astrocytes and microglia, through intercellular signaling pathways, especially in the context of BACE1 deficiency, thereby facilitating Aβ clearance and maintaining homeostasis.

## Figures and Tables

**Fig. (1):**
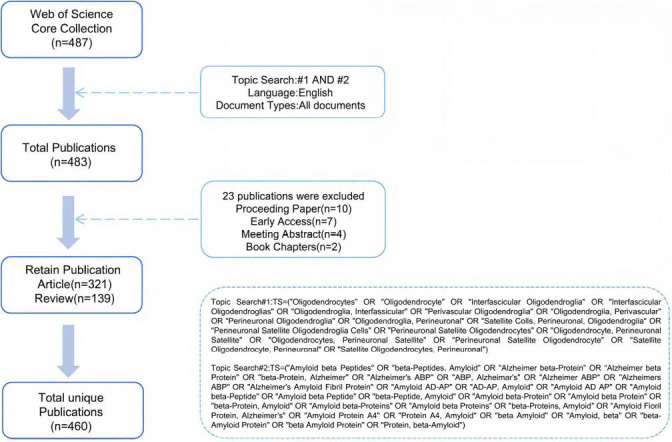
Flowchart of data collection and study design.

## LIST OF ABBREVIATIONS

AD: Alzheimer's Disease
Aβ: Amyloid-beta
OPCs: Oligodendrocyte Precursor Cells
OLs: Oligodendrocytes
CNS: Central Nervous System
NFTs: Neurofibrillary Tangles
WOS: Web of Science
APP: Amyloid Precursor Protein
BACE1: Beta Secretase
sAD: Sporadic Alzheimer's Disease
iPSC: Induced Pluripotent Stem Cell
PSEN1: Presenilin-1
PSEN2: Presenilin-2
NCSTN: Nicas trin
nSMase: Neutral Sphingomyelinase
MBP: Myelin Basic Protein
Itgb1: Integrin β1
